# Association of the Systemic Immune-Inflammation Index with Ascending Aortic Diameter and Dilatation: A Retrospective Cross-Sectional Study

**DOI:** 10.3390/medicina62101892

**Published:** 2026-09-30

**Authors:** Selin Yılmaz, Mert Nahir, Ataç Çelik

**Affiliations:** 1 Department of Anatomy, Tokat Gaziosmanpaşa University, 60250 Tokat, Turkey; 2 Department of Cardiology, Tokat Gaziosmanpaşa University, 60250 Tokat, Turkey

**Keywords:** aortic aneurysm, thoracic, aorta, thoracic, inflammation, computed tomography angiography, neutrophils, blood platelets

## Abstract

*Background and Objectives*: Complete blood count-derived inflammatory indices are inexpensive and widely available, but data on their relationship with ascending aortic size are limited. This study examined the association between the systemic immune-inflammation index (SII) and ascending aortic diameter, and compared these indices according to the presence of ascending aortic dilatation (AAD) defined by a 40 mm threshold. *Materials and Methods*: In this single-centre retrospective cross-sectional study, 251 patients aged 18 years or older (mean age, 53.1 ± 16.7 years; range, 18–95 years; 160 men) who underwent thoracic computed tomography angiography were grouped by maximum tubular ascending aortic diameter (<40 mm, *n* = 158; ≥40 mm, *n* = 93). Inference for the primary linear model was based on heteroscedasticity-consistent (HC3) standard errors. Height and weight were not recorded, so the models could not be adjusted for body size. *Results*: In unadjusted comparisons, platelet and neutrophil counts, creatinine and all five inflammatory indices were nominally higher in the dilated group (all *p* < 0.05), whereas C-reactive protein did not differ. In the primary adjusted model, each 100-unit increase in SII was associated with a 0.59 mm greater ascending aortic diameter (HC3 95% CI 0.28 to 0.90; β = 0.197; *p* < 0.001), approximately 1.6 mm across the interquartile range of SII; the model explained 26.9% of the variance. Higher SII was also associated with AAD in the secondary logistic model (odds ratio per 100 units, 1.34; 95% CI 1.17 to 1.53; *p* < 0.001). Discrimination was weak (area under the curve for SII, 0.658; 95% CI 0.590 to 0.726); the sample-derived cut-off gave a positive likelihood ratio of 1.38, and its 2.5–97.5th percentile interval on bootstrap internal validation was 305 to 579. None of the 75 correlations survived false discovery rate adjustment, and the SII-by-sex interaction was not significant (*p* = 0.621). *Conclusions*: Higher SII was associated with a greater ascending aortic diameter and with the presence of AAD after adjustment for the measured covariates; because body size could not be accounted for, residual confounding cannot be excluded. The adjusted difference was modest and discrimination was weak. These findings do not support the use of SII as a standalone diagnostic marker for ascending aortic dilatation.

## 1. Introduction

Ascending aortic dilatation is usually asymptomatic and is most often detected incidentally on imaging performed for other indications [1]. Its clinical importance derives from the natural history of the dilated aorta: acute aortic syndromes carry a high mortality, and population-based data indicate that 48.6% of patients with incident type A aortic dissection die before hospital assessment, with an in-hospital mortality of approximately 27% among those who reach hospital [2,3]. Aortic diameter is not a fixed quantity; it varies with age, sex, height and body surface area [1,4,5,6], and traditional cardiovascular risk factors such as diastolic blood pressure, atherosclerosis and lipid levels are also associated with it [7]. Accordingly, current guidelines describe the dilated ascending aorta in terms of absolute diameter thresholds [8] and of dimensions indexed to body size [9].

The pathogenesis of ascending aortic dilatation is multifactorial, and inflammatory and immune-mediated mechanisms have attracted increasing attention. Inflammatory involvement of the aortic wall, as seen in giant cell arteritis, syphilitic aortitis and atherosclerosis, may contribute to aneurysm formation [10], and circulating inflammatory biomarkers have been related to thoracic and abdominal aortic pathology [11,12]. An association between ascending aortic diameter and high-sensitivity C-reactive protein has also been reported [13].

Composite indices derived from the complete blood count offer an inexpensive and universally available alternative to dedicated inflammatory assays. The systemic immune-inflammation index (SII), calculated as (neutrophil count × platelet count)/lymphocyte count, was first described in 2014 [14] and has since been evaluated in malignancies and in cardiovascular disease [15,16]. Related indices include the neutrophil-to-lymphocyte ratio [17], the platelet-to-lymphocyte ratio [18], the pan-immune-inflammation value, which has been associated with abdominal aortic calcification [19,20], and the systemic inflammation response index [21]. Data on these indices in ascending aortic disease, however, remain limited.

This study examined the association between the systemic immune-inflammation index and the maximum tubular ascending aortic diameter in adults undergoing thoracic computed tomography angiography. Analyses of ascending aortic dilatation defined by an absolute 40 mm threshold were secondary, and analyses of the other inflammatory indices, the aortic diameters measured at other anatomical levels and the sex-stratified comparisons were exploratory.

## 2. Materials and Methods

### 2.1. Study Design and Setting

**Figure 1:**
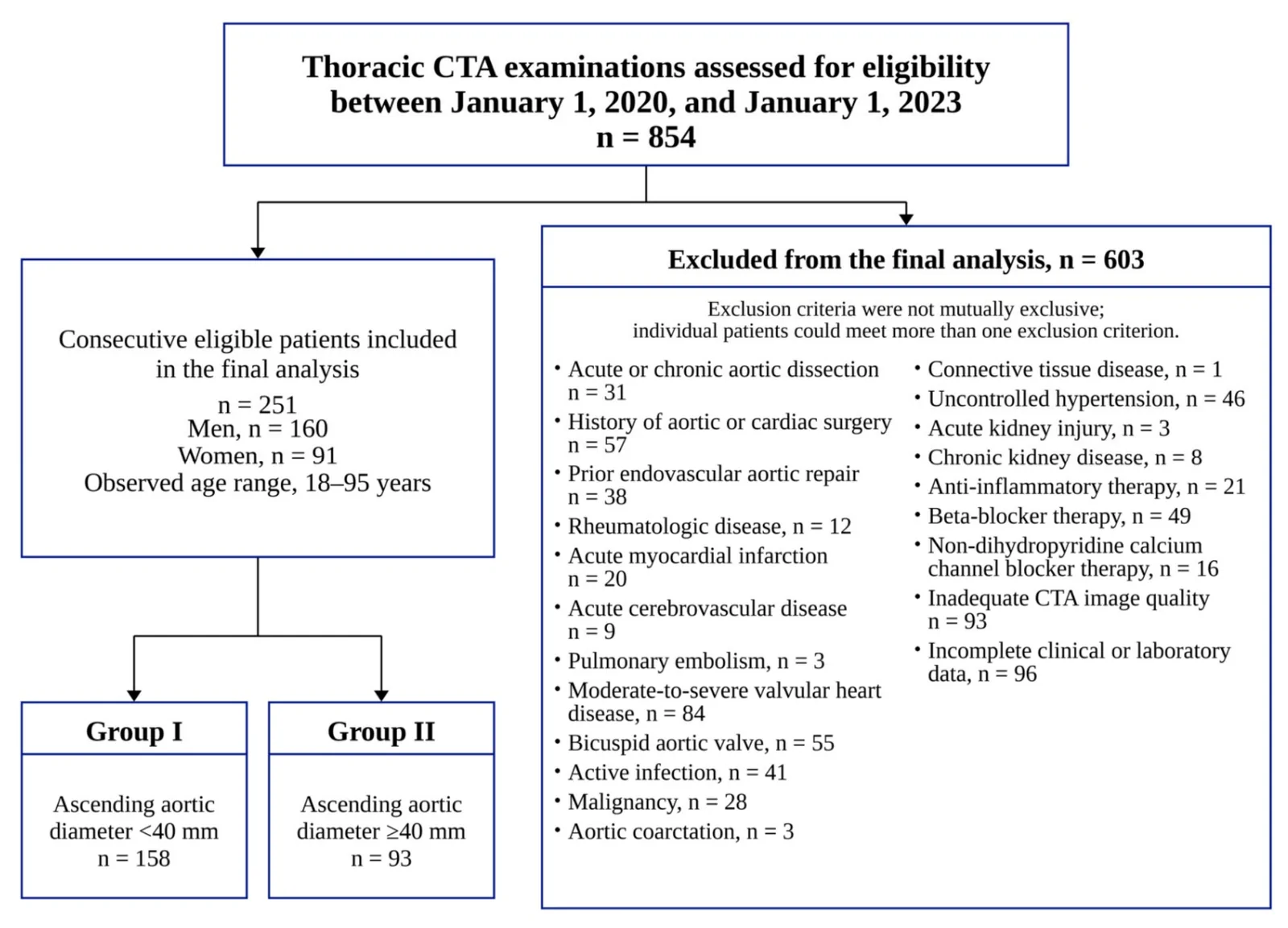
Flowchart of patient selection. CTA, computed tomography angiography. Exclusion criteria were not mutually exclusive; individual patients could meet more than one criterion, and therefore the sum of the listed exclusions exceeds the total number of excluded patients.

This was a single-centre, retrospective, cross-sectional study based on thoracic computed tomography angiography (CTA) examinations performed in the radiology department of our institution between 1 January 2020 and 1 January 2023. Eligibility was assessed at the level of the CTA examination: consecutive examinations of patients aged 18 years or older performed during this period were screened, and all examinations meeting the eligibility criteria were included without matching or predefined group balancing. Each included examination corresponded to a unique patient, and no patient contributed more than one examination to the analysis. The selection process is shown in Figure 1. Because all eligible patients during the study period were included, no a priori sample-size calculation was performed. The study was conducted in accordance with the Declaration of Helsinki and was approved by the Human Research Ethics Committee of Tokat Gaziosmanpaşa University (protocol code 24-KAEK-026, 15 February 2024); the requirement for written informed consent was waived by the committee owing to the retrospective design.

### 2.2. Eligibility Criteria

Patients were eligible if they were aged 18 years or older, had a thoracic CTA examination of diagnostic quality, and had a complete blood count and biochemistry panel obtained on the same day as the CTA examination and before the administration of intravenous contrast material.

Exclusion criteria were defined in advance and comprised conditions or treatments expected to influence either aortic dimensions or circulating inflammatory indices: acute or chronic aortic dissection; a history of aortic or cardiac surgery; prior endovascular aortic repair; aortic coarctation; bicuspid aortic valve; moderate-to-severe valvular heart disease; heritable connective tissue disease; rheumatologic disease; acute myocardial infarction; acute cerebrovascular disease; pulmonary embolism; active infection; malignancy; uncontrolled hypertension; acute kidney injury; and chronic kidney disease. Patients receiving beta-blocker therapy, non-dihydropyridine calcium channel blocker therapy, or anti-inflammatory therapy were also excluded, as were patients with inadequate CTA image quality or incomplete clinical or laboratory data. Because the exclusion criteria were not mutually exclusive, individual patients could meet more than one criterion, and the sum of the listed exclusions therefore exceeds the total number of excluded patients.

The medication-related exclusions warrant explanation. Beta-blockers and non-dihydropyridine calcium channel blockers are preferentially prescribed to patients with known aortic dilatation, hypertension, or coronary disease, so that retaining these patients could have introduced confounding by indication; in addition, the haemodynamic effects of these agents on heart rate and aortic wall stress may influence aortic dimensions. Patients receiving anti-inflammatory therapy were excluded because such treatment directly alters leukocyte and platelet counts and hence the derived indices. Anti-inflammatory therapy was defined as ongoing systemic anti-inflammatory or immunosuppressive treatment at the time of the CTA examination, comprising systemic corticosteroids, immunosuppressive agents and biologic agents; intermittent or as-needed non-steroidal anti-inflammatory drug use was not an exclusion criterion. Statins were deliberately not treated as anti-inflammatory therapy for the purposes of exclusion; statin use was instead recorded and examined as a covariate in sensitivity analyses. We acknowledge that these exclusions, together with the exclusion of chronic kidney disease, uncontrolled hypertension, and moderate-to-severe valvular heart disease, removed a substantial proportion of patients with established, treated cardiovascular disease; the implications for generalisability are considered in the Section 4.

### 2.3. Clinical Definitions and Outcomes

The primary outcome was the maximum diameter of the tubular ascending aorta, analysed as a continuous variable in millimetres. As a secondary, categorical outcome, ascending aortic dilatation (AAD) was defined operationally as a maximum tubular ascending aortic diameter of 40 mm or greater, and patients were assigned to Group I (<40 mm) or Group II (≥40 mm). This threshold is consistent with the 2022 ACC/AHA aortic disease guideline, in which an ascending aortic diameter of 4.0–4.4 cm is designated “dilated” and a diameter of ≥4.5 cm “aneurysmal” [8]. The 2024 ESC guideline additionally describes body surface area-indexed dimensions for the assessment of aortic size [9]; because height and weight were not recorded in the source records, body size-indexed classification could not be evaluated, and an absolute threshold was used throughout. The threshold denotes dilatation; it is not used interchangeably with thoracic aortic aneurysm, nor as an indication for treatment.

Uncontrolled hypertension was defined as documented hypertension with persistent office systolic blood pressure ≥ 140 mmHg and/or diastolic blood pressure ≥ 90 mmHg at or near the time of the CTA examination, confirmed by a repeat office measurement or by available home or ambulatory blood pressure measurements, irrespective of treatment status [22]. Active infection was defined as a clinically documented ongoing infection at the time of CTA, supported by compatible signs or symptoms and, where available, microbiological or imaging evidence and/or ongoing antimicrobial treatment. Acute kidney injury was defined according to KDIGO criteria as an increase in serum creatinine of ≥0.3 mg/dL within 48 h or to ≥1.5 times the baseline value within the preceding 7 days, with urine-output criteria applied where available [23]. Chronic kidney disease was defined according to the KDIGO 2012 criteria as abnormalities of kidney structure or function present for at least 3 months, including an estimated glomerular filtration rate < 60 mL/min/1.73 m^2^ or other markers of kidney damage [24].

Hyperlipidaemia was defined as a documented diagnosis of dyslipidaemia in the medical record. Statin use was recorded separately as any ongoing statin prescription at the time of the CTA examination, irrespective of the documented indication; consequently, a small number of patients receiving a statin for secondary prevention were not coded as having hyperlipidaemia. Both variables were recorded because statins have pleiotropic anti-inflammatory effects that may influence complete blood count-derived indices, and both were examined in sensitivity analyses.

Height and weight were not recorded in the source records. Body surface area- or height-indexed aortic diameters could therefore not be calculated, and the multivariable models could not be adjusted for body size. The implications of this limitation are addressed in the Section 4.

### 2.4. Imaging and Measurement Reliability

All examinations were performed with the patient supine, following intravenous administration of 80 mL of non-ionic iodinated contrast material at an injection rate of 4 mL/s, followed by a 40 mL saline flush. Electrocardiographically gated CTA was performed on a GE Healthcare Optima CT 660 scanner (GE Healthcare Medical Systems, Bangalore, India) with the following parameters: scan time, 2 s; tube current–time product, 49 mAs; field of view, 163 mm; slice thickness, 0.6 mm; and tube voltage, 100 kV.

Aortic diameters were measured at five anatomical levels using multiplanar reconstruction: the aortic root, the sinus of Valsalva, the sinotubular junction, the tubular ascending aorta, and the aorta at the diaphragmatic level. All diameters were measured from inner edge to inner edge in planes perpendicular to the long axis of the vessel. The sinus of Valsalva diameter was recorded as the maximum sinus-to-sinus measurement, and the maximum tubular ascending aortic diameter was determined in a plane perpendicular to the vessel axis.

All examinations were evaluated by the first observer, and these measurements were used in all analyses. Interobserver reliability was assessed in a subset of 40 patients selected using the random number function of Microsoft Excel (version 2019; Microsoft Corp., Redmond, WA, USA), without replacement. Both observers were blinded to the patients’ laboratory findings and inflammatory marker results. In a separate session, the second observer, additionally blinded to the first observer’s measurements, independently remeasured the maximum tubular ascending aortic diameter. The intraclass correlation coefficient (ICC) was calculated from the paired measurements obtained before consensus, using a two-way random-effects, absolute-agreement, single-measurement specification [ICC(2,1)] with 95% confidence intervals [25]; the single-measurement form was used because a single observer’s measurement was analysed for each patient. Discrepant measurements were subsequently reviewed by consensus. The mean difference between observers was calculated as the first observer’s measurement minus the second observer’s, with a 95% confidence interval from the paired *t*-test, and the 95% limits of agreement were calculated as the mean difference ± 1.96 times the standard deviation of the paired differences. Reliability was assessed only for the maximum tubular ascending aortic diameter; the remaining four levels, which were used only in exploratory correlation analyses, were measured by a single observer.

### 2.5. Laboratory Measurements

Complete blood count and biochemistry results obtained on the day of the CTA examination, before contrast administration, were used for all patients. Complete blood counts were analysed on a Sysmex XN-1000 automated haematology analyser (Sysmex Corp., Kobe, Japan) and biochemical parameters on a Cobas c501 automated biochemistry analyser (Roche Diagnostics International AG, Rotkreuz, Switzerland).

The parameters recorded were white blood cell count, neutrophil, lymphocyte and monocyte counts, haemoglobin concentration and platelet count; blood urea nitrogen and creatinine; sodium, potassium and chloride; aspartate aminotransferase and alanine aminotransferase; and C-reactive protein. Composite indices were calculated from the cell counts as follows: SII = (neutrophil count × platelet count)/lymphocyte count; NLR = neutrophil count/lymphocyte count; PLR = platelet count/lymphocyte count; PIV = (neutrophil count × monocyte count × platelet count)/lymphocyte count; and SIRI = (neutrophil count × monocyte count)/lymphocyte count. All indices were computed from the recorded cell counts at full numerical precision rather than from stored rounded values.

### 2.6. Statistical Analysis

The primary analysis was the association between SII and the maximum tubular ascending aortic diameter, analysed as a continuous outcome in a multivariable linear model. The association between SII and the presence of AAD, defined by the 40 mm threshold, was a secondary adjusted analysis. Analyses involving NLR, PLR, PIV and SIRI, the aortic diameters measured at the other four anatomical levels, the receiver operating characteristic (ROC) analyses, and the sex-stratified comparisons were exploratory. No written statistical analysis plan predated the analysis; the primary exposure, outcome and covariate set were selected before modelling, and all other analyses are reported as secondary or exploratory.

Categorical variables are presented as counts and percentages, and continuous variables as mean ± standard deviation and/or median (Q1–Q3), as appropriate. Normality was assessed with the Lilliefors-corrected Kolmogorov–Smirnov test. Continuous variables were compared between groups with the independent-samples *t*-test when normality was satisfied in both groups and with the Mann–Whitney U test otherwise. Age did not satisfy normality and was therefore compared with the Mann–Whitney U test in the overall population and in both sex-based subgroups, and is presented as both mean ± standard deviation and median (Q1–Q3). Categorical variables were compared with Pearson’s chi-square test when all expected cell counts were 5 or greater and with Fisher’s exact test otherwise.

The primary adjusted analysis was a multivariable linear regression of ascending aortic diameter on SII, adjusted for age, sex, hypertension, current smoking, diabetes mellitus, coronary artery disease and creatinine, with SII modelled per 100-unit increase. Model assumptions were examined using residual plots, the Shapiro–Wilk test for residual normality, the Breusch–Pagan test for homoscedasticity, and variance inflation factors for multicollinearity. Because the Breusch–Pagan test indicated heteroscedasticity, inference for this model is based on heteroscedasticity-consistent (HC3) standard errors, and the overall model was assessed with a heteroscedasticity-robust Wald test. Results obtained with conventional standard errors are provided in Supplementary Table S3 for comparison. HC3 standard errors address non-constant error variance only; they do not address confounding or selection. The SII-by-sex interaction was tested and, being non-significant, was not retained in the final main-effects model.

Sensitivity analyses repeated the primary model after substituting the natural logarithm of SII, and after additional adjustment for statin use, for hyperlipidaemia, and for C-reactive protein; all used HC3 standard errors. Hyperlipidaemia and statin use were not entered into the same model because, as coded, every participant with hyperlipidaemia was receiving a statin, and their joint inclusion produced variance inflation factors above 8.

As a secondary adjusted analysis, multivariable logistic regression was used to model the presence of AAD using the same covariate set, with SII modelled per 100-unit increase. Model fit was assessed with the Hosmer–Lemeshow test and discrimination with the C statistic; both refer to apparent performance in the same sample and were not externally validated.

Correlations between aortic diameters and inflammatory indices were examined with Spearman’s rank correlation. Only the 75 correlations were adjusted for multiple comparisons, using the Benjamini–Hochberg false discovery rate procedure across the full set of 75 tests; for these, a q value below 0.05 was considered statistically significant. No multiplicity adjustment was applied to the group comparisons, the sex-stratified analyses, the ROC analyses or the sensitivity analyses; their *p* values are nominal, and these results are reported descriptively as hypothesis-generating findings. The primary adjusted model was not subject to multiplicity adjustment. For all analyses other than these, a two-sided *p* value below 0.05 was considered statistically significant. No imputation was performed, because patients with incomplete clinical or laboratory data were excluded.

The ability of the inflammatory indices to discriminate participants with and without AAD was assessed with ROC analysis, and areas under the curve are reported with DeLong 95% confidence intervals. Cut-off values were derived with the Youden index and are reported as the midpoint between the two adjacent observed marker values. Because these cut-offs were derived and evaluated in the same sample, their apparent sensitivity and specificity are optimistic. This was quantified by bootstrap internal validation with 1000 resamples: within each resample the Youden cut-off was re-derived and evaluated both in that resample and in the original sample, and the mean difference (optimism) was subtracted from the apparent estimates. The 2.5th and 97.5th percentiles of the resampled cut-offs are reported to describe cut-off stability, and predictive values and likelihood ratios at the cut-off are given in Supplementary Table S2. No external validation was performed, and these cut-offs are not proposed for clinical use.

Analyses were performed with IBM SPSS Statistics version 27.0 (IBM Corp., Armonk, NY, USA) and R version 4.4.3 (R Foundation for Statistical Computing, Vienna, Austria). The HC3 standard errors were computed with the sandwich package (version 3.1-1), the Breusch–Pagan test with lmtest (version 0.9-40), and variance inflation factors with car (version 3.1-3); the heteroscedasticity-robust Wald test, the DeLong confidence intervals, the intraclass correlation coefficient and the bootstrap internal validation were implemented directly in base R. A fixed random seed (20240101) was used to ensure reproducibility of the bootstrap procedure.

## 3. Results

### 3.1. Patient Selection and Characteristics

Of the 854 thoracic CTA examinations screened, 603 were excluded and 251 entered the analysis; the selection process and the reasons for exclusion are shown in Figure 1. Of these, 160 (63.7%) were men and 91 (36.3%) were women. The mean age was 53.1 ± 16.7 years (median, 56 years [Q1–Q3, 42–65]; observed range, 18–95 years). One hundred and fifty-eight patients were assigned to Group I (ascending aortic diameter < 40 mm) and 93 to Group II (≥40 mm).

Demographic characteristics and comorbidities according to sex are presented in Table 1. Age and the prevalence of hypertension, diabetes mellitus, coronary artery disease and cerebrovascular disease did not differ significantly between men and women. Current smoking was substantially more frequent among men than among women (66.9% vs. 12.1%; *p* < 0.001), and both hyperlipidaemia (32.5% vs. 20.9%; *p* = 0.049) and statin use (35.6% vs. 23.1%; *p* = 0.039) were nominally more frequent among men.

Demographic characteristics according to ascending aortic diameter are presented in Table 2. In unadjusted comparisons, participants in Group II were older (median, 61 years [52–70] vs. 50 years [35–63]; *p* < 0.001) and had a higher prevalence of hypertension (63.4% vs. 39.9%; *p* < 0.001) than those in Group I. Statin use was more frequent in Group II (39.8% vs. 25.9%; *p* = 0.022), whereas the difference in hyperlipidaemia did not reach statistical significance (35.5% vs. 24.1%; *p* = 0.052). No significant differences were observed for diabetes mellitus, coronary artery disease, cerebrovascular disease or current smoking. The sex-stratified panels of Table 2 are exploratory, and all *p* values in Table 1 and Table 2 are nominal.

### 3.2. Aortic Measurements and Measurement Reliability

The mean maximum tubular ascending aortic diameter was 34.8 ± 3.5 mm in Group I and 47.3 ± 4.8 mm in Group II. Interobserver reliability for this measurement, assessed in 40 patients, was excellent [ICC(2,1) = 0.96; 95% CI 0.93 to 0.98]. The paired *t*-test did not detect a statistically significant mean difference between observers (mean difference, 0.25 mm; 95% CI −0.24 to 0.74; *p* = 0.308), and the 95% limits of agreement were −2.75 to 3.25 mm.

### 3.3. Laboratory Findings and Inflammatory Indices

Laboratory findings according to ascending aortic diameter are presented in Table 3, and the sex-stratified panels in Supplementary Table S1. In the overall population, platelet count, neutrophil count, creatinine, NLR, PLR, SII, PIV and SIRI were nominally higher in Group II in unadjusted comparisons (all *p* < 0.05), whereas white blood cell count and C-reactive protein did not differ significantly between the groups.

The median SII was 426.82 (Q1–Q3, 293.42–552.08) in Group I and 524.48 (394.72–650.63) in Group II (*p* < 0.001). In the exploratory sex-stratified comparisons, the median SII was 453.32 versus 501.69 in women (*p* = 0.132) and 395.49 versus 531.79 in men (*p* < 0.001). Because the SII-by-sex interaction was not statistically significant (*p* = 0.621), these subgroup differences should not be interpreted as evidence of a sex-specific association. C-reactive protein was nominally higher in Group II among women (*p* = 0.043) but not in the overall population or among men; this isolated subgroup finding was not adjusted for multiple comparisons and should be interpreted cautiously.

### 3.4. Correlation Analyses

Spearman correlations between aortic diameters at the five anatomical levels and the five inflammatory indices are presented in Table 4 with nominal *p* values and Benjamini–Hochberg false discovery rate-adjusted q values. Fourteen of the 75 correlations were nominally significant, but none remained statistically significant after false discovery rate adjustment in the overall population or in the female and male subgroups (smallest q = 0.122). These analyses are exploratory.

**Table 4:** Spearman correlations between aortic measurements and systemic inflammatory markers (exploratory).

Marker	Aortic Root	Sinus of Valsalva	Sinotubular Junction	Ascending Aorta	Diaphragmatic Level
**AllParticipants**					
SII	−0.132/0.037/0.199	0.012/0.851/0.945	0.002/0.973/0.973	0.146/0.020/0.138	−0.163/0.009/0.122
NLR	−0.039/0.541/0.736	0.071/0.261/0.544	0.048/0.447/0.722	0.163/0.010/0.122	−0.063/0.319/0.616
PLR	−0.167/0.008/0.122	−0.105/0.098/0.349	−0.073/0.248/0.531	0.062/0.328/0.616	−0.096/0.130/0.410
PIV	−0.113/0.074/0.291	0.080/0.207/0.511	0.041/0.522/0.736	0.108/0.087/0.325	−0.144/0.022/0.138
SIRI	−0.045/0.481/0.722	0.117/0.063/0.291	0.062/0.328/0.616	0.114/0.072/0.291	−0.075/0.236/0.521
**Female Participants**					
SII	−0.199/0.058/0.291	−0.018/0.863/0.945	−0.012/0.910/0.945	0.041/0.697/0.871	−0.244/0.020/0.138
NLR	−0.149/0.159/0.443	0.051/0.633/0.804	0.065/0.543/0.736	0.077/0.471/0.722	−0.159/0.131/0.410
PLR	−0.130/0.219/0.511	−0.085/0.423/0.721	−0.069/0.517/0.736	−0.117/0.271/0.549	−0.128/0.225/0.511
PIV	−0.273/0.009/0.122	−0.012/0.910/0.945	−0.025/0.811/0.945	0.039/0.712/0.876	−0.257/0.014/0.138
SIRI	−0.240/0.022/0.138	0.011/0.917/0.945	0.006/0.956/0.969	0.061/0.565/0.743	−0.229/0.029/0.166
**Male Participants**					
SII	−0.056/0.479/0.722	0.070/0.381/0.680	0.048/0.549/0.736	0.236/0.003/0.122	−0.097/0.225/0.511
NLR	−0.016/0.841/0.945	0.053/0.503/0.736	0.011/0.893/0.945	0.186/0.018/0.138	−0.056/0.480/0.722
PLR	−0.116/0.143/0.427	−0.057/0.475/0.722	−0.019/0.807/0.945	0.214/0.006/0.122	−0.018/0.824/0.945
PIV	−0.039/0.626/0.804	0.122/0.123/0.410	0.066/0.404/0.705	0.144/0.068/0.291	−0.115/0.148/0.427
SIRI	−0.008/0.920/0.945	0.107/0.179/0.481	0.028/0.729/0.882	0.100/0.210/0.511	−0.074/0.350/0.640

Data are presented as Spearman’s correlation coefficient/nominal *p* value/Benjamini–Hochberg false discovery rate-adjusted q value (r/*p*/q). The false discovery rate adjustment was applied across all 75 correlations. None of the correlations remained statistically significant after adjustment (smallest q = 0.122).

### 3.5. Primary Adjusted Analysis

The Breusch–Pagan test indicated heteroscedasticity (*p* < 0.001), and inference for the primary linear model is therefore based on HC3 standard errors. Variance inflation factors were all below 1.9, indicating no meaningful multicollinearity, and the residuals deviated modestly from normality (Shapiro–Wilk *p* < 0.001; skewness 0.42).

In the primary adjusted model, higher SII (B = 0.591 mm per 100-unit increase; HC3 95% CI 0.281 to 0.901; β = 0.197; *p* < 0.001) and older age (B = 0.142 mm per year; HC3 95% CI 0.086 to 0.198; β = 0.327; *p* < 0.001) were associated with a greater ascending aortic diameter after adjustment for the covariates listed in Table 5. Expressed across the observed interquartile range of SII (339 to 605), the SII coefficient corresponds to a difference of approximately 1.6 mm in ascending aortic diameter. The model explained 26.9% of the variance (R^2^ = 0.269; adjusted R^2^ = 0.245; heteroscedasticity-robust Wald F(8, 242) = 10.22; *p* < 0.001). Height and weight were not recorded, so the model could not be adjusted for body size. The complete results are presented in Table 5, and the corresponding results obtained with conventional standard errors in Supplementary Table S3.

**Table 5:** Multivariable linear regression analysis for ascending aortic diameter.

Variable	B	HC3 95% CI for B	β	*p* Value
SII (per 100-unit increase)	0.591	0.281 to 0.901	0.197	<0.001
Age (per year)	0.142	0.086 to 0.198	0.327	<0.001
Male sex	1.665	−0.494 to 3.823	0.111	0.130
Hypertension	1.724	−0.295 to 3.742	0.119	0.094
Current smoking	−0.094	−1.992 to 1.805	−0.006	0.923
Diabetes mellitus	0.716	−1.452 to 2.884	0.044	0.516
Coronary artery disease	0.983	−1.844 to 3.810	0.054	0.494
Creatinine (per mg/dL)	1.626	−4.229 to 7.481	0.040	0.585

Model summary: R^2^ = 0.269; adjusted R^2^ = 0.245; *n* = 251. Because the Breusch–Pagan test indicated heteroscedasticity (*p* < 0.001), all confidence intervals and *p* values are based on heteroscedasticity-consistent (HC3) standard errors, and the overall model was assessed with a heteroscedasticity-robust Wald test [F(8, 242) = 10.22, *p* < 0.001]. Results obtained with conventional standard errors are provided in Supplementary Table S3. The dependent variable was ascending aortic diameter in millimetres. Female sex was coded as 0 and male sex as 1; hypertension, current smoking, diabetes mellitus, and coronary artery disease were coded as 0 = absent and 1 = present. Height and weight were not available, so the model could not be adjusted for body size. B indicates the unstandardised regression coefficient; β, the standardised regression coefficient; CI, confidence interval; and SII, systemic immune-inflammation index.

### 3.6. Sensitivity Analyses

The association between SII and ascending aortic diameter was consistent across sensitivity analyses. Substituting the natural logarithm of SII yielded a comparable result (B = 3.017; HC3 95% CI 1.578 to 4.456; β = 0.208; *p* < 0.001). Additional adjustment for statin use (B = 0.592 per 100 units; HC3 95% CI 0.281 to 0.902; *p* < 0.001), for hyperlipidaemia (B = 0.593; HC3 95% CI 0.281 to 0.905; *p* < 0.001), or for C-reactive protein (B = 0.608; HC3 95% CI 0.271 to 0.944; *p* < 0.001) did not materially alter the estimate. Hyperlipidaemia and statin use were not entered into the same model because their joint inclusion produced variance inflation factors above 8.

SII was not correlated with age (ρ = −0.003; *p* = 0.965) and did not differ between men and women (median, 451.5 vs. 461.5; *p* = 0.238) or between statin users and non-users (median, 458.5 vs. 454.8; *p* = 0.735).

### 3.7. Secondary Adjusted Analysis

In the multivariable logistic regression model for the presence of AAD, adjusted for the same covariates, higher SII (odds ratio per 100-unit increase, 1.34; 95% CI 1.17 to 1.53; *p* < 0.001) and older age (odds ratio per year, 1.04; 95% CI 1.01 to 1.06; *p* = 0.003) were associated with AAD, whereas male sex (odds ratio 1.72; 95% CI 0.77 to 3.81; *p* = 0.184) and hypertension (odds ratio 1.66; 95% CI 0.85 to 3.23; *p* = 0.138) were not. The Hosmer–Lemeshow test did not indicate evidence of poor fit (*p* = 0.309). The C statistic of the full model was 0.760 (95% CI 0.701 to 0.819) compared with 0.658 for SII alone; both refer to apparent discrimination in the same sample and were not externally validated. The complete results are presented in Table 6.

### 3.8. Discriminative Performance and Internal Validation

**Figure 2:**
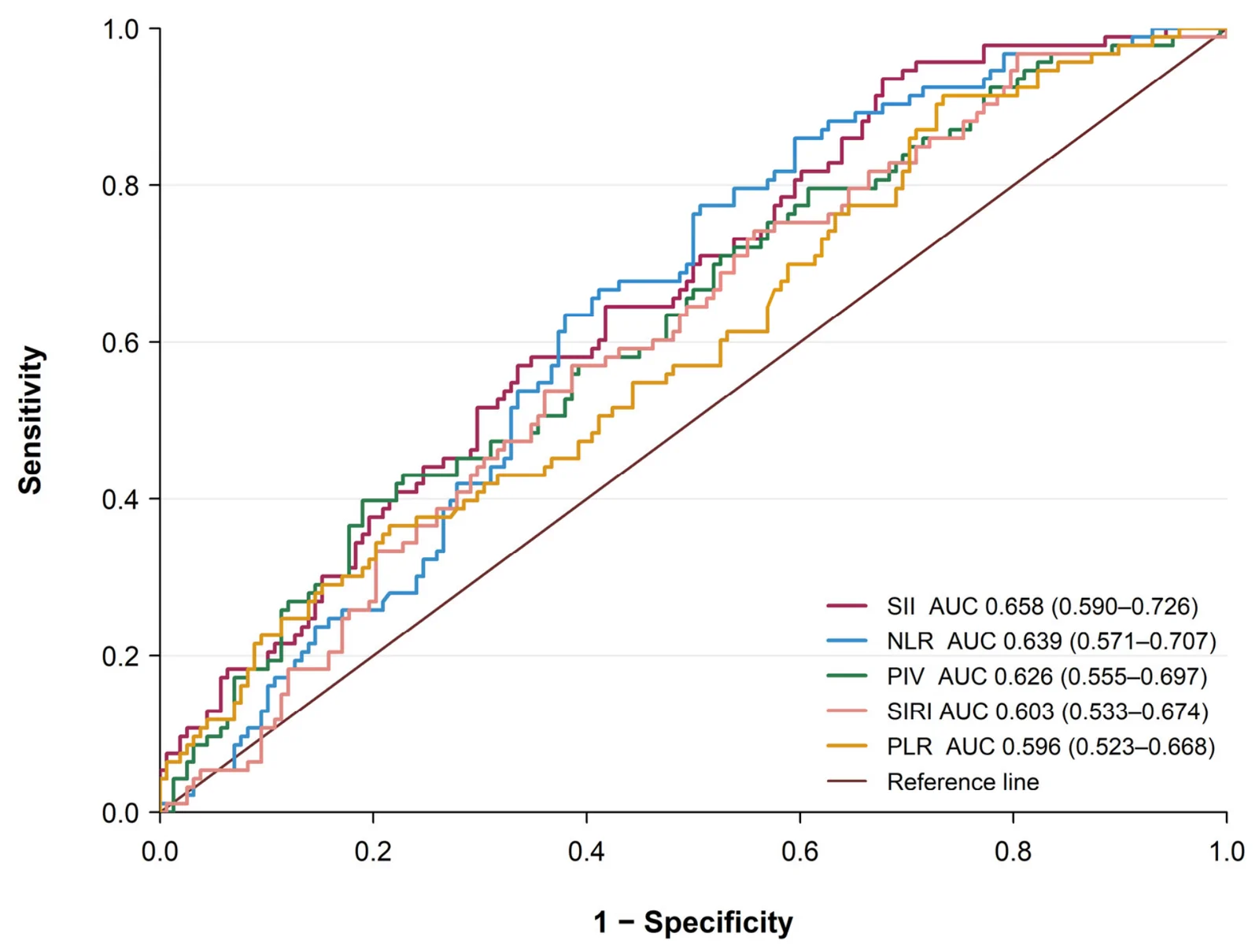
Receiver operating characteristic curves of systemic inflammatory markers for discriminating participants with and without ascending aortic dilatation. Areas under the curve with 95% confidence intervals (DeLong method) are shown in the legend. All curves represent apparent discrimination in the study sample; cut-off values were derived with the Youden index in the same dataset. Internal validation by bootstrap resampling and performance measures at each cut-off are provided in Supplementary Table S2. NLR, neutrophil-to-lymphocyte ratio; PIV, pan-immune-inflammation value; PLR, platelet-to-lymphocyte ratio; SII, systemic immune-inflammation index; and SIRI, systemic inflammation response index.

The areas under the curve for discriminating participants with and without AAD were 0.658 (95% CI 0.590–0.726) for SII, 0.639 (0.571–0.707) for NLR, 0.626 (0.555–0.697) for PIV, 0.603 (0.533–0.674) for SIRI and 0.596 (0.523–0.668) for PLR (Figure 2). The sample-derived Youden cut-off for SII was 323.15, with an apparent sensitivity of 93.5% and specificity of 32.3%; the corresponding positive likelihood ratio was 1.38, the negative likelihood ratio 0.20, and overall accuracy 55.0%. The remaining cut-offs were 1.63 for NLR, 379.30 for PIV, 0.85 for SIRI and 78.40 for PLR.

On bootstrap internal validation with 1000 resamples, the 2.5–97.5th percentile interval of the resampled SII cut-offs was 304.8 to 578.8, and the optimism-corrected sensitivity and specificity were 90.4% and 30.4%. The corresponding percentile intervals were 1.3 to 1.9 for NLR, 137.1 to 393.0 for PIV, 0.5 to 1.4 for SIRI and 77.8 to 161.6 for PLR. Optimism in the area under the curve was small for all five indices, as expected for single markers whose coefficients are not estimated; this procedure addresses the optimism arising from selection of the cut-off and does not quantify other sources of uncertainty. The complete results, including predictive values and likelihood ratios at each cut-off, are presented in Supplementary Table S2. These cut-offs were derived and evaluated in the same sample, were not validated externally, and are not proposed for clinical use.

## 4. Discussion

In this single-centre cross-sectional study of 251 patients undergoing thoracic computed tomography angiography, complete blood count-derived inflammatory indices were nominally higher in participants with ascending aortic dilatation in unadjusted comparisons, and higher SII was associated with a greater ascending aortic diameter in the primary adjusted model and with the presence of AAD in the secondary logistic model. The magnitude of the association was small: across the interquartile range of SII, the adjusted difference in diameter was approximately 1.6 mm. None of the 75 exploratory correlations survived false discovery rate adjustment, the discriminative performance of SII was weak, and the SII-by-sex interaction was not significant.

Ascending aortic dilatation was defined here by an absolute diameter of 40 mm or greater. This threshold denotes dilatation and should not be read as equivalent to thoracic aortic aneurysm, which current guidelines place at a larger diameter, nor as an indication for intervention. It should also be noted that the 2024 ESC guideline considers an ascending aorta exceeding 22 mm/m^2^ of body surface area to be dilated and, for the aortic root, cites sex-specific absolute values (>40 mm in men and >36 mm in women) [9]. Because height and weight were not recorded, the indexed definition could not be applied in this cohort, and a single absolute threshold of 40 mm was used in both sexes; some women classified here as non-dilated might therefore be classified as dilated under sex-specific or indexed criteria. The groups compared here are therefore defined by absolute size, and the classification of individual participants close to the threshold will depend in part on body size.

Inflammatory and immune-mediated mechanisms have attracted growing attention in the pathogenesis of ascending aortic disease, and inflammatory infiltration of the aortic wall has been related to reduced biomechanical strength in type A dissection and ascending aneurysm [11]. Neutrophils and lymphocytes secrete mediators that can contribute to degeneration of the vascular wall, and chronic inflammation has an established role in cardiovascular disease more broadly [26]. Against this background, complete blood count-derived indices are attractive because they are inexpensive, reproducible and available from a test that is already performed; their value, however, depends on whether they carry information beyond that available from age, sex and conventional risk factors.

Our unadjusted findings are numerically concordant with earlier work. Güngör et al. reported a median NLR of 2.04 in patients with ascending aortic dilatation versus 1.78 in controls [13]; the corresponding values in our cohort were 2.00 and 1.67. That study also reported an association with high-sensitivity C-reactive protein, whereas standard C-reactive protein did not differ significantly in our overall population. A plausible explanation is the lower analytical sensitivity of the standard assay at the concentrations relevant here; a nominally higher C-reactive protein was observed among women with AAD, but this isolated subgroup finding was not adjusted for multiple comparisons and should be interpreted cautiously.

Higher SII has also been reported in other vascular phenotypes: in abdominal aortic calcification [27], in isolated coronary artery ectasia [28], and in unruptured intracranial fusiform aneurysms, in which higher SII was associated with aneurysmal wall enhancement [29]. Studies in aortic aneurysm have more often addressed postoperative outcome than the presence of dilatation itself; SII has also been associated with survival after endovascular repair of abdominal aortic aneurysms [30]. The present findings extend this literature to the ascending aorta measured as a continuous variable, in a setting where the outcome, the population and the scale of measurement differ from those of the earlier reports.

Two further observations are relevant to the interpretation of the association. SII was not correlated with age in the unadjusted analysis (ρ = −0.003; *p* = 0.965), and it did not differ detectably between statin users and non-users (median 458.5 vs. 454.8; *p* = 0.735). These are descriptive findings; they do not by themselves exclude a confounding role for age or statin use, and the principal evidence on this point comes from the adjusted model and the sensitivity analyses. Neither observation establishes that the association is independent of other measured or unmeasured factors. The most important of these is body size: aortic diameter increases with height and body surface area, and adiposity is associated with higher complete blood count-derived inflammatory indices, so unmeasured body size is a plausible source of residual confounding that could alter both the magnitude and the direction of the observed association. Adjustment for sex captures only part of this variation. The findings reported here should therefore be read as an association after adjustment for the measured covariates, not as an association independent of body size.

The cross-sectional design imposes a further limit on interpretation. It cannot be determined whether systemic inflammation precedes aortic dilatation, follows it, or shares common determinants with it; a dilated aorta may itself elicit a low-grade systemic inflammatory response. For the same reason, the adjusted coefficient should be read as a difference in diameter between individuals with different SII values at a single point in time, not as a rate of growth or as a prognostic estimate for an individual patient.

It is also worth stating plainly how the adjusted model and the correlation analyses relate to one another. The adjusted model and the Spearman correlations answer different questions: the former estimates the association between SII and diameter holding the covariates constant, the latter the unadjusted monotonic association at each aortic level, with a multiplicity penalty applied across 75 tests. The failure of any correlation to survive that penalty is not evidence that the adjusted model is invalid, nor is it a detail that can be set aside; both results are reported, and together they indicate an association that is statistically evident in the adjusted analysis but modest in magnitude and not detectable as a simple bivariate correlation once multiplicity is taken into account.

The discriminative performance of all five indices was weak, with areas under the curve between 0.60 and 0.66, and the sample-derived cut-offs performed poorly in clinical terms. For SII, the cut-off of 323.15 combined a high sensitivity (93.5%) with a low specificity (32.3%), yielding a positive likelihood ratio of 1.38 and an overall accuracy of 55.0%. A positive likelihood ratio of this magnitude moves the post-test probability very little, so a result above this threshold carries little diagnostic information. The cut-offs were moreover derived and evaluated in the same sample: on bootstrap internal validation the 2.5–97.5th percentile interval of the resampled SII cut-offs was 304.8 to 578.8, and the optimism-corrected sensitivity and specificity were 90.4% and 30.4%. Taken together, these findings do not support the use of SII, or of any of the other indices examined, as a standalone diagnostic marker for ascending aortic dilatation, and the cut-offs reported here should not be applied in clinical practice.

With regard to the sex-stratified analyses, men constituted the majority of the study population. Sex differences in thoracic aortic disease are well recognised, and although the incidence of ascending aortic dilatation is lower in women, affected women have been reported to face higher risks of dissection, rupture and death [31]. Several nominal differences were observed in our exploratory sex-stratified analyses, most conspicuously a significant SII difference among men but not among women. These should not be interpreted as evidence of a sex-specific association: the SII-by-sex interaction was not statistically significant (*p* = 0.621), the subgroup analyses were not adjusted for multiple comparisons, and the female subgroup was small (27 women with AAD). Differences in nominal significance between the subgroups may reflect differences in precision; the interaction analysis did not provide evidence of effect modification by sex, and it does not establish that the association is identical in women and men.

The study population was highly selected, and this deserves explicit comment. Of 854 thoracic CTA examinations screened, 251 (29%) entered the analysis after the application of broad exclusion criteria, and the participants were patients referred for CTA rather than a sample of the general population. The medication-related exclusions in particular shaped the cohort: because beta-blockers and non-dihydropyridine calcium channel blockers are frequently prescribed to patients with known aortic dilatation, hypertension or coronary disease, their exclusion (together with the exclusion of chronic kidney disease, uncontrolled hypertension and moderate-to-severe valvular heart disease) is likely to have removed many patients with established, treated cardiovascular disease, and may therefore have shifted the cohort toward untreated, incidentally detected dilatation. These exclusions were intended to reduce potential confounding by treatment and by concomitant inflammatory disease, but they introduce a different selection, one that adjustment for statin use does not resolve. Many patients had undergone echocardiography or chest radiography before thoracic CTA, and some examinations may therefore have been requested because aortic enlargement was already suspected. The indications for CTA were not, however, systematically recorded, so referral bias cannot be quantified, and the direction of its effect on the observed association cannot be determined. The prevalence of AAD in this cohort (37.1%) is accordingly far higher than in the general population, which is why the predictive values reported in Supplementary Table S2 are not transportable to other settings.

Several limitations should be considered. First and most importantly, height and weight were not recorded, so aortic diameters could not be indexed to body surface area or height and the multivariable models could not be adjusted for body size. Because body size is a major determinant of aortic diameter and is also related to inflammatory indices, residual confounding by body size cannot be excluded and could alter the magnitude or direction of the observed association; for the same reason the absolute 40 mm threshold classifies participants without regard to their body habitus, which is particularly relevant to the sex-stratified analyses.

Second, the study was conducted at a single centre in a selected population, as described above, which limits generalisability to the broader population of patients with ascending aortic dilatation. Third, the cross-sectional design precludes any inference about temporality or direction, and the inflammatory indices were derived from a single blood sample obtained on the day of CTA; these indices are subject to substantial within-individual biological variability, and repeated measurements were not available. Interobserver reliability was assessed only for the maximum tubular ascending aortic diameter and only in a subset of 40 patients, whereas the remaining four anatomical levels used in the exploratory correlation analyses were measured by a single observer.

Fourth, a large number of exploratory analyses were performed. Only the 75 correlations in Table 4 were adjusted for multiple comparisons; the group comparisons, sex-stratified analyses and ROC analyses were not, and their *p* values are nominal. The receiver operating characteristic cut-offs were derived and evaluated in the same sample and were not validated externally. For these reasons the secondary and exploratory findings reported here are hypothesis-generating and should not be used to guide diagnosis or surveillance. Finally, the sample size limited the precision of the subgroup analyses.

## 5. Conclusions

In this single-centre cross-sectional study, complete blood count-derived inflammatory indices were higher in participants with ascending aortic dilatation in unadjusted comparisons, and higher SII was associated with a greater ascending aortic diameter and with the presence of AAD after adjustment for the measured covariates; adjustment for body size was not possible and residual confounding remains possible. The adjusted difference was modest, corresponding to approximately 1.6 mm across the interquartile range of SII. None of the 75 correlations remained significant after false discovery rate correction, and the non-significant SII-by-sex interaction does not support a sex-specific association. The limited discriminative performance, with a positive likelihood ratio of 1.38 at the sample-derived cut-off, does not support the use of SII as a standalone diagnostic marker for ascending aortic dilatation. Prospective, multicentre studies with body size-indexed aortic measurements and external validation of any proposed threshold are required before these inexpensive indices can be considered as adjunctive markers in the evaluation of ascending aortic dilatation.

## Figures and Tables

**Table 1:** Demographic characteristics and comorbidities according to sex.

Variable	Total (*n* = 251)	Female (*n* = 91, 36.3%)	Male (*n* = 160, 63.7%)	*p* Value
Age, years, mean ± SD	53.1 ± 16.7	51.2 ± 17.4	54.1 ± 16.3	0.257
Age, years, median (Q1–Q3)	56 (42–65)	52 (36–64)	56 (44–66)	0.257
Hypertension, *n* (%)	122 (48.6)	38 (41.8)	84 (52.5)	0.102
Diabetes mellitus, *n* (%)	67 (26.7)	19 (20.9)	48 (30.0)	0.116
Coronary artery disease, *n* (%)	49 (19.5)	13 (14.3)	36 (22.5)	0.114
Cerebrovascular disease, *n* (%)	24 (9.6)	8 (8.8)	16 (10.0)	0.754
Current smoking, *n* (%)	118 (47.0)	11 (12.1)	107 (66.9)	<0.001
Hyperlipidaemia, *n* (%)	71 (28.3)	19 (20.9)	52 (32.5)	0.049
Statin use, *n* (%)	78 (31.1)	21 (23.1)	57 (35.6)	0.039

Data are presented as mean ± standard deviation, median (Q1–Q3), or *n* (%). Age was compared using the Mann–Whitney U test; categorical variables were compared using Pearson’s chi-square test or Fisher’s exact test, as appropriate. All *p* values are nominal and were not adjusted for multiple comparisons.

**Table 2:** Comparison of demographic characteristics and comorbidities according to ascending aortic diameter.

Variable	Group I (Ascending Aortic Diameter < 40 mm)	Group II (Ascending Aortic Diameter ≥ 40 mm)	*p* Value
**All Participants**	***n* = 158**	***n* = 93**	
Age, years, mean ± SD	49.3 ± 17.3	59.4 ± 13.4	<0.001
Age, years, median (Q1–Q3)	50 (35–63)	61 (52–70)	
Hypertension, *n* (%)	63 (39.9)	59 (63.4)	<0.001
Diabetes mellitus, *n* (%)	38 (24.1)	29 (31.2)	0.217
Coronary artery disease, *n* (%)	25 (15.8)	24 (25.8)	0.054
Cerebrovascular disease, *n* (%)	11 (7.0)	13 (14.0)	0.068
Current smoking, *n* (%)	77 (48.7)	41 (44.1)	0.476
Hyperlipidaemia, *n* (%)	38 (24.1)	33 (35.5)	0.052
Statin use, *n* (%)	41 (25.9)	37 (39.8)	0.022
**Female Participants**	***n* = 64**	***n* = 27**	
Age, years, mean ± SD	47.2 ± 17.4	60.7 ± 13.4	0.002
Age, years, median (Q1–Q3)	49 (30–62)	61 (53–70)	
Hypertension, *n* (%)	21 (32.8)	17 (63.0)	0.008
Diabetes mellitus, *n* (%)	11 (17.2)	8 (29.6)	0.182
Coronary artery disease, *n* (%)	6 (9.4)	7 (25.9)	0.052
Cerebrovascular disease, *n* (%)	3 (4.7)	5 (18.5)	0.047
Current smoking, *n* (%)	7 (10.9)	4 (14.8)	0.726
Hyperlipidaemia, *n* (%)	11 (17.2)	8 (29.6)	0.182
Statin use, *n* (%)	11 (17.2)	10 (37.0)	0.040
**Male Participants**	***n* = 94**	***n* = 66**	
Age, years, mean ± SD	50.7 ± 17.2	58.9 ± 13.5	0.002
Age, years, median (Q1–Q3)	52 (39–63)	60 (50–70)	
Hypertension, *n* (%)	42 (44.7)	42 (63.6)	0.018
Diabetes mellitus, *n* (%)	27 (28.7)	21 (31.8)	0.674
Coronary artery disease, *n* (%)	19 (20.2)	17 (25.8)	0.408
Cerebrovascular disease, *n* (%)	8 (8.5)	8 (12.1)	0.454
Current smoking, *n* (%)	70 (74.5)	37 (56.1)	0.015
Hyperlipidaemia, *n* (%)	27 (28.7)	25 (37.9)	0.224
Statin use, *n* (%)	30 (31.9)	27 (40.9)	0.242

Group I, ascending aortic diameter < 40 mm; Group II, ascending aortic diameter ≥ 40 mm. Data are presented as mean ± standard deviation, median (Q1–Q3), or *n* (%). Age did not satisfy normality and was compared using the Mann–Whitney U test in the overall population and in both sex-based subgroups. Categorical variables were compared using Pearson’s chi-square test or Fisher’s exact test, as appropriate. All *p* values are nominal and were not adjusted for multiple comparisons; the sex-stratified panels are exploratory.

**Table 3:** Laboratory results and inflammatory indices according to ascending aortic diameter (all participants).

Variable	Group I (*n* = 158)	Group II (*n* = 93)	*p* Value
WBC (10^3^/µL)	7.29 (6.34–8.82)	7.79 (6.88–8.73)	0.061
Haemoglobin (g/dL)	14.10 (12.60–15.07)	14.20 (12.70–15.30)	0.816
Platelet count (10^3^/µL)	235.50 (211.00–291.75)	257.00 (227.00–316.00)	0.009
Lymphocytes (10^3^/µL)	2.43 (1.93–2.98)	2.28 (1.91–2.78)	0.173
Neutrophils (10^3^/µL)	4.01 (3.21–5.05)	4.64 (4.02–5.36)	<0.001
Monocytes (10^3^/µL)	0.57 (0.45–0.72)	0.58 (0.46–0.71)	0.839
Creatinine (mg/dL)	0.80 (0.69–0.91)	0.88 (0.78–0.98)	0.005
BUN (mg/dL)	13.80 (11.40–16.65)	15.10 (12.10–19.05)	0.166
Sodium (mmol/L)	140.00 (138.00–142.00)	141.00 (138.00–142.00)	0.629
Potassium (mmol/L)	4.4 ± 0.4	4.4 ± 0.4	0.829
Chloride (mmol/L)	103.8 ± 2.6	103.8 ± 2.5	0.961
AST (U/L)	19.00 (16.00–23.00)	18.30 (16.00–22.00)	0.576
ALT (U/L)	18.10 (14.05–26.00)	17.30 (13.50–23.00)	0.623
CRP (mg/L)	2.16 (1.12–4.00)	2.95 (1.31–4.25)	0.108
NLR	1.67 (1.27–2.24)	2.00 (1.65–2.44)	<0.001
PLR	103.32 (77.03–127.80)	110.73 (90.73–151.13)	0.012
SII	426.82 (293.42–552.08)	524.48 (394.72–650.63)	<0.001
PIV	238.12 (161.64–341.83)	301.65 (212.65–458.46)	<0.001
SIRI	0.92 (0.67–1.39)	1.16 (0.83–1.54)	0.006

Data are presented as median (Q1–Q3), except for potassium and chloride, which satisfied normality and are presented as mean ± standard deviation. Normality was assessed with the Lilliefors-corrected Kolmogorov–Smirnov test; groups were compared with the Mann–Whitney U test or the independent-samples *t*-test accordingly. All *p* values are nominal and were not adjusted for multiple comparisons. Sex-stratified results are given in Supplementary Table S1. ALT, alanine aminotransferase; AST, aspartate aminotransferase; BUN, blood urea nitrogen; CRP, C-reactive protein; NLR, neutrophil-to-lymphocyte ratio; PIV, pan-immune-inflammation value; PLR, platelet-to-lymphocyte ratio; SII, systemic immune-inflammation index; SIRI, systemic inflammation response index; and WBC, white blood cell count.

**Table 6:** Multivariable logistic regression analysis for the presence of ascending aortic dilatation (secondary adjusted analysis).

Variable	OR	95% CI	*p* Value
SII (per 100-unit increase)	1.341	1.174 to 1.531	<0.001
Age (per year)	1.036	1.012 to 1.060	0.003
Male sex	1.717	0.774 to 3.806	0.184
Hypertension	1.656	0.850 to 3.228	0.138
Current smoking	0.815	0.409 to 1.627	0.562
Diabetes mellitus	0.970	0.497 to 1.896	0.930
Coronary artery disease	0.912	0.434 to 1.915	0.807
Creatinine (per mg/dL)	1.667	0.252 to 11.017	0.596

Model summary: Omnibus χ^2^ = 50.58, df = 8, *p* < 0.001; Nagelkerke R^2^ = 0.249; and events per variable = 11.6. The Hosmer–Lemeshow test did not indicate evidence of poor fit (*p* = 0.309). The C statistic was 0.760 (95% CI 0.701 to 0.819) and reflects apparent discrimination in the same sample; it was not externally validated. The dependent variable was the presence of ascending aortic dilatation (ascending aortic diameter ≥ 40 mm). Female sex was coded as 0 and male sex as 1; hypertension, current smoking, diabetes mellitus, and coronary artery disease were coded as 0 = absent and 1 = present. Confidence intervals are Wald intervals. Height and weight were not available, so the model could not be adjusted for body size. CI, confidence interval; OR, odds ratio; and SII, systemic immune-inflammation index.

## Data Availability

The de-identified dataset analysed in this study is available from the corresponding author upon reasonable request.

## Supplementary Materials

The following supporting information can be downloaded at: https://www.mdpi.com/article/10.3390/medicina62101892/s1, Table S1: Laboratory results and inflammatory indices according to ascending aortic diameter, overall and by sex; Table S2: Discriminative performance of inflammatory indices for ascending aortic dilatation, with bootstrap internal validation and performance measures at the sample-derived cut-off; Table S3: Conventional and HC3 robust standard errors and the resulting inference for the primary linear model.

## Abbreviations

The following abbreviations are used in this manuscript:

AAD	ascending aortic dilatation
ACC/AHA	American College of Cardiology/American Heart Association
ALT	alanine aminotransferase
AST	aspartate aminotransferase
AUC	area under the curve
BUN	blood urea nitrogen
CI	confidence interval
CRP	C-reactive protein
CTA	computed tomography angiography
ESC	European Society of Cardiology
HC3	heteroscedasticity-consistent standard errors (type 3)
ICC	intraclass correlation coefficient
KDIGO	Kidney Disease: Improving Global Outcomes
NLR	neutrophil-to-lymphocyte ratio
OR	odds ratio
PIV	pan-immune-inflammation value
PLR	platelet-to-lymphocyte ratio
ROC	receiver operating characteristic
SD	standard deviation
SII	systemic immune-inflammation index
SIRI	systemic inflammation response index
WBC	white blood cell count
