# Supplementary material for: Association of the Systemic Immune-Inflammation Index with Ascending Aortic Diameter and Dilatation: A Retrospective Cross-Sectional Study
Source: Medicina (Kaunas). 2026 Sep 30;62(10):1892. doi: 10.3390/medicina62101892 (PMC13706893; doi:10.3390/medicina62101892)
Supplement: Supplementary file 1 [file medicina-62-01892-s001.zip › Table_S1.pdf]

Table S1. Laboratory Results and Inflammatory Indices According to Ascending Aortic Diameter, Overall and by Sex

Variable	Group I, mean ± SD	Group I, median (Q1–Q3)	Group II, mean ± SD	Group II, median (Q1–Q3)	P value
**ALL PARTICIPANTS**	**n = 158**		**n = 93**		
WBC (10^3/µL)	7.6 ± 2.1	7.29 (6.34–8.82)	8.0 ± 1.6	7.79 (6.88–8.73)	.061 **
Hemoglobin (g/dL)	14.0 ± 1.9	14.10 (12.60–15.07)	13.9 ± 1.9	14.20 (12.70–15.30)	.816 **
Platelet count (10^3/µL)	247.5 ± 56.1	235.50 (211.00–291.75)	274.2 ± 72.1	257.00 (227.00–316.00)	.009 **
Lymphocytes (10^3/µL)	2.6 ± 0.9	2.43 (1.93–2.98)	2.4 ± 0.7	2.28 (1.91–2.78)	.173 **
Neutrophils (10^3/µL)	4.2 ± 1.5	4.01 (3.21–5.05)	4.8 ± 1.2	4.64 (4.02–5.36)	<.001 **
Monocytes (10^3/µL)	0.6 ± 0.2	0.57 (0.45–0.72)	0.6 ± 0.2	0.58 (0.46–0.71)	.839 **
Creatinine (mg/dL)	0.8 ± 0.2	0.80 (0.69–0.91)	0.9 ± 0.2	0.88 (0.78–0.98)	.005 **
BUN (mg/dL)	14.7 ± 4.9	13.80 (11.40–16.65)	15.4 ± 5.4	15.10 (12.10–19.05)	.166 **
Sodium (mmol/L)	140.2 ± 2.8	140.00 (138.00–142.00)	140.3 ± 2.8	141.00 (138.00–142.00)	.629 **
Potassium (mmol/L)	4.4 ± 0.4	4.37 (4.18–4.61)	4.4 ± 0.4	4.36 (4.12–4.64)	.829 *
Chloride (mmol/L)	103.8 ± 2.6	104.20 (101.50–105.60)	103.8 ± 2.5	103.80 (102.30–105.50)	.961 *
AST (U/L)	20.3 ± 6.5	19.00 (16.00–23.00)	20.8 ± 10.1	18.30 (16.00–22.00)	.576 **
ALT (U/L)	20.6 ± 10.3	18.10 (14.05–26.00)	21.6 ± 14.8	17.30 (13.50–23.00)	.623 **
CRP (mg/L)	2.9 ± 2.6	2.16 (1.12–4.00)	3.4 ± 2.9	2.95 (1.31–4.25)	.108 **
NLR	1.8 ± 0.9	1.67 (1.27–2.24)	2.1 ± 0.8	2.00 (1.65–2.44)	<.001 **
PLR	106.5 ± 37.4	103.32 (77.03–127.80)	123.3 ± 46.4	110.73 (90.73–151.13)	.012 **
SII	446.5 ± 208.5	426.82 (293.42–552.08)	584.2 ± 268.0	524.48 (394.72–650.63)	<.001 **
PIV	275.1 ± 181.1	238.12 (161.64–341.83)	350.7 ± 197.6	301.65 (212.65–458.46)	<.001 **
SIRI	1.1 ± 0.7	0.92 (0.67–1.39)	1.3 ± 0.7	1.16 (0.83–1.54)	.006 **
**FEMALE PARTICIPANTS**	**n = 64**		**n = 27**		
WBC (10^3/µL)	7.3 ± 1.8	7.20 (5.97–8.59)	8.2 ± 1.7	8.36 (7.06–8.82)	.018 *
Hemoglobin (g/dL)	12.8 ± 1.5	12.80 (12.10–13.80)	13.0 ± 1.6	12.90 (12.25–14.10)	.676 *
Platelet count (10^3/µL)	269.5 ± 58.8	264.50 (223.50–315.50)	293.4 ± 74.9	296.00 (232.50–330.00)	.107 *
Lymphocytes (10^3/µL)	2.4 ± 0.7	2.33 (1.93–2.82)	2.7 ± 0.9	2.43 (1.91–3.18)	.159 *
Neutrophils (10^3/µL)	4.1 ± 1.3	4.01 (3.22–5.08)	4.8 ± 1.1	4.86 (4.19–5.39)	.027 *
Monocytes (10^3/µL)	0.5 ± 0.2	0.54 (0.45–0.62)	0.5 ± 0.2	0.52 (0.45–0.68)	.961 *
Creatinine (mg/dL)	0.7 ± 0.1	0.70 (0.62–0.77)	0.8 ± 0.1	0.77 (0.68–0.85)	.121 *
BUN (mg/dL)	13.4 ± 4.7	12.50 (10.29–14.62)	15.2 ± 6.7	13.40 (11.75–17.20)	.143 **
Sodium (mmol/L)	140.3 ± 2.7	140.50 (138.00–142.00)	140.3 ± 3.2	141.00 (138.00–142.00)	.716 **
Potassium (mmol/L)	4.4 ± 0.4	4.37 (4.21–4.58)	4.4 ± 0.3	4.40 (4.21–4.57)	.847 *
Chloride (mmol/L)	103.9 ± 2.6	103.75 (101.92–105.82)	103.8 ± 2.6	104.10 (102.40–105.10)	.945 *
AST (U/L)	19.2 ± 5.8	18.00 (14.90–21.30)	17.7 ± 5.8	16.20 (14.20–20.85)	.189 **
ALT (U/L)	18.6 ± 8.7	16.85 (12.95–21.10)	17.6 ± 10.4	15.90 (11.55–19.25)	.352 **
CRP (mg/L)	3.1 ± 3.0	2.66 (1.18–3.98)	4.5 ± 4.0	3.45 (2.20–4.95)	.043 **
NLR	1.8 ± 0.7	1.77 (1.39–2.14)	2.0 ± 0.9	1.81 (1.39–2.41)	.364 **
PLR	117.6 ± 32.0	115.27 (97.07–135.87)	122.2 ± 48.5	108.39 (85.55–162.67)	.597 *
SII	481.3 ± 186.3	453.32 (359.60–596.06)	588.5 ± 296.5	501.69 (403.58–699.41)	.132 **
PIV	267.8 ± 135.7	243.05 (180.49–360.21)	338.9 ± 235.0	285.82 (176.17–434.41)	.316 **
SIRI	1.0 ± 0.5	0.90 (0.68–1.20)	1.2 ± 0.7	0.97 (0.68–1.44)	.501 **
**MALE PARTICIPANTS**	**n = 94**		**n = 66**		
WBC (10^3/µL)	7.9 ± 2.2	7.46 (6.38–8.84)	7.8 ± 1.6	7.62 (6.84–8.59)	.534 **
Hemoglobin (g/dL)	14.7 ± 1.7	14.90 (13.90–15.80)	14.3 ± 1.9	14.65 (13.32–15.67)	.301 **
Platelet count (10^3/µL)	232.5 ± 49.0	223.50 (202.25–260.75)	266.3 ± 70.0	255.00 (217.25–296.00)	.002 **
Lymphocytes (10^3/µL)	2.6 ± 1.0	2.45 (1.94–3.20)	2.3 ± 0.6	2.21 (1.93–2.66)	.026 **
Neutrophils (10^3/µL)	4.3 ± 1.7	4.00 (3.19–5.02)	4.7 ± 1.2	4.59 (4.00–5.35)	.004 **
Monocytes (10^3/µL)	0.6 ± 0.3	0.62 (0.46–0.78)	0.6 ± 0.2	0.62 (0.47–0.75)	.666 **
Creatinine (mg/dL)	0.9 ± 0.2	0.88 (0.78–1.01)	0.9 ± 0.2	0.92 (0.82–1.00)	.167 **
BUN (mg/dL)	15.6 ± 4.8	14.80 (12.25–17.00)	15.5 ± 4.8	15.20 (12.30–19.28)	.853 **
Sodium (mmol/L)	140.2 ± 2.9	140.00 (138.00–142.00)	140.3 ± 2.6	141.00 (138.25–142.00)	.727 **
Potassium (mmol/L)	4.4 ± 0.4	4.38 (4.16–4.61)	4.4 ± 0.4	4.33 (4.08–4.66)	.673 *
Chloride (mmol/L)	103.8 ± 2.7	104.20 (101.32–105.50)	103.8 ± 2.4	103.60 (102.15–105.60)	.973 *
AST (U/L)	21.0 ± 6.9	19.80 (16.50–23.43)	22.0 ± 11.3	20.00 (16.10–22.85)	.837 **
ALT (U/L)	21.9 ± 11.1	19.60 (14.87–27.15)	23.3 ± 16.0	18.65 (13.62–27.38)	.695 **
CRP (mg/L)	2.8 ± 2.3	2.04 (1.12–4.07)	2.9 ± 2.1	2.34 (1.09–4.18)	.467 **
NLR	1.9 ± 1.0	1.58 (1.18–2.27)	2.2 ± 0.8	2.04 (1.69–2.42)	<.001 **
PLR	99.0 ± 39.1	92.09 (72.19–119.73)	123.7 ± 45.9	112.41 (92.14–149.32)	<.001 **
SII	422.7 ± 220.2	395.49 (254.35–531.93)	582.5 ± 257.9	531.79 (392.01–642.04)	<.001 **
PIV	280.2 ± 206.9	229.03 (137.45–332.46)	355.5 ± 182.0	311.59 (218.69–456.38)	.001 **
SIRI	1.2 ± 0.9	0.94 (0.67–1.44)	1.3 ± 0.6	1.31 (0.88–1.58)	.017 **

Data are presented as mean ± standard deviation and median (Q1–Q3). * independent-samples t-test; ** Mann–Whitney U test. Normality was assessed with the Lilliefors-corrected Kolmogorov–Smirnov test. All p values are nominal; the sex-stratified panels are exploratory.

Abbreviations as in Table 3.
