# Supplementary material for: Association of the Systemic Immune-Inflammation Index with Ascending Aortic Diameter and Dilatation: A Retrospective Cross-Sectional Study
Source: Medicina (Kaunas). 2026 Sep 30;62(10):1892. doi: 10.3390/medicina62101892 (PMC13706893; doi:10.3390/medicina62101892)
Supplement: Supplementary file 1 [file medicina-62-01892-s001.zip › Table_S2.pdf]

Table S2. Discriminative Performance of Inflammatory Indices for Ascending Aortic Dilatation, With Bootstrap Internal Validation and Performance Measures at the Sample-Derived Cut-off

**Marker**	**AUC (95% CI)**	**AUC, corr.**	**Cut-off**	**Bootstrap range**	**Sens., % (corr.)**	**Spec., % (corr.)**	**PPV, %**	**NPV, %**	**LR+**	**LR−**	**Acc., %**
SII	0.658 (0.590 to 0.726)	0.656	323.15	304.8–578.8	93.5 (90.4)	32.3 (30.4)	44.8	89.5	1.38	0.20	55.0
NLR	0.639 (0.571 to 0.707)	0.641	1.63	1.3–1.9	77.4 (75.0)	49.4 (47.9)	47.4	78.8	1.53	0.46	59.8
PIV	0.626 (0.555 to 0.697)	0.626	379.30	137.1–393.0	39.8 (36.5)	81.0 (78.9)	55.2	69.6	2.10	0.74	65.7
SIRI	0.603 (0.533 to 0.674)	0.603	0.85	0.5–1.4	74.2 (70.7)	44.3 (42.3)	43.9	74.5	1.33	0.58	55.4
PLR	0.596 (0.523 to 0.668)	0.595	78.40	77.8–161.6	91.4 (88.1)	26.6 (24.7)	42.3	84.0	1.24	0.32	50.6

Areas under the curve and their 95% confidence intervals were obtained with the DeLong method. Cut-off values were derived with the Youden index and are reported as the midpoint between the two adjacent observed marker values. Because the cut-offs were derived and evaluated in the same sample, a bootstrap procedure with 1,000 resamples was used: within each resample the Youden cut-off was re-derived and evaluated both in that resample and in the original sample, and the mean difference (optimism) was subtracted from the apparent estimates. Optimism-corrected values are given in parentheses, and the bootstrap range is the 2.5th–97.5th percentile of the resampled cut-offs. Predictive values reflect the observed prevalence of ascending aortic dilatation in this sample (37.1%) and are not transportable to populations with a different prevalence. No external validation was performed, and these cut-offs are not proposed for clinical use.

Acc., accuracy; AUC, area under the curve; corr., optimism-corrected; LR+, positive likelihood ratio; LR−, negative likelihood ratio; NLR, neutrophil-to-lymphocyte ratio; NPV, negative predictive value; PIV, pan-immune-inflammation value; PLR, platelet-to-lymphocyte ratio; PPV, positive predictive value; Sens., sensitivity; SII, systemic immune-inflammation index; SIRI, systemic inflammation response index; Spec., specificity.
