# Supplementary material for: Association of the Systemic Immune-Inflammation Index with Ascending Aortic Diameter and Dilatation: A Retrospective Cross-Sectional Study
Source: Medicina (Kaunas). 2026 Sep 30;62(10):1892. doi: 10.3390/medicina62101892 (PMC13706893; doi:10.3390/medicina62101892)
Supplement: Supplementary file 1 [file medicina-62-01892-s001.zip › Table_S3.pdf]

Table S3: Conventional and HC3 robust standard errors and the resulting inference for the primary linear model.

Variable	B	OLS 95% CI	OLS P value	HC3 95% CI	HC3 P value
SII (per 100-unit increase)	0.591	0.260 to 0.922	<.001	0.281 to 0.901	<.001
Age (per year)	0.142	0.082 to 0.202	<.001	0.086 to 0.198	<.001
Male sex	1.665	-0.549 to 3.878	.140	-0.494 to 3.823	.130
Hypertension	1.724	-0.176 to 3.623	.075	-0.295 to 3.742	.094
Current smoking	-0.094	-2.028 to 1.841	.924	-1.992 to 1.805	.923
Diabetes mellitus	0.716	-1.227 to 2.659	.469	-1.452 to 2.884	.516
Coronary artery disease	0.983	-1.173 to 3.138	.370	-1.844 to 3.810	.494
Creatinine (per mg/dL)	1.626	-3.782 to 7.035	.554	-4.229 to 7.481	.585

B, the unstandardised regression coefficient, is identical under both methods; only the standard errors and the quantities derived from them differ. The model is the one reported in Table 5.

CI, confidence interval; HC3, heteroscedasticity-consistent standard errors of type 3; OLS, ordinary least squares; SII, systemic immune-inflammation index.
